# Supplementary material for: Are carbon emissions trading and green financial instruments synergistic? -Comprehensive quantitative research based on content analysis
Source: PLoS One. 2024 Mar 7;19(3):e0298601. doi: 10.1371/journal.pone.0298601 (PMC10919601; doi:10.1371/journal.pone.0298601)
Supplement: S1 Data — (ZIP) [file pone.0298601.s001.zip › Data/地级市Z_F工作报告.zip!/╕╩╦α/╞╜┴╣╩╨/└┤╫╘╣1⁄2╓┌║┼í╛┬φ┐╦╩2╛▌═°í┐_2014.docx]

各位代表：

现在，我代表市人民政府向大会作工作报告，请予审议。并请政协委员和列席人员提出意见。

2013年工作回顾

刚刚过去的一年，在市委的坚强领导下，在市人大、市政协的监督支持下，我们团结依靠全市人民，认真贯彻落实党的十八大和十八届二中、三中全会精神，牢牢把握稳中求进、好中求快的总基调和提速进位、重点突破、改革创新、抓实见效的总体要求，锐意进取，攻坚克难，忠实履行人民赋予的职责，扎实做好各方面工作，全市经济社会保持了平稳较快发展的良好态势。预计地区生产总值完成335亿元，比上年增长10%；地方财政收入22.62亿元，同口径增长18.9%，财政支出134.97亿元，增长8.9%；城镇居民人均可支配收入17677元，农民人均纯收入4847元，分别增长14%和15%。

一年来，我们做了以下主要工作：

（一）全力稳增长，经济实现平稳较快发展

面对市场环境变化和经济下行压力，我们及时采取应对措施，进一步强化责任目标管理，建立经济形势监测预警和月调度、季分析制度，加强对重点工作、重点行业的指导协调和调度管理，促使主要经济指标稳中有增、回升向好。

大力实施项目带动行动，围绕落实全省“3341”项目工程，以“十大工程”为引领，狠抓项目谋划论证和衔接争取，千方百计扩大招商引资，与浙江精工、江苏金浦、平顶山煤机公司、西蒙集团、秦宝牧业等知名企业洽谈对接，签约和引进了一批煤化工、装备制造、牛果产业开发等项目，组建招商引资项目落地办公室，强化跟踪服务，招商引资到位资金316.3亿元，增长62.2%。全年实施500万元以上项目997项，其中亿元以上项目175项，固定资产投资总额达到545亿元，拉动生产总值增长3.9个百分点。

针对煤电市场需求减弱、工业经济增长乏力的严峻形势，建立工业经济联席会议制度，设立工业发展专项资金，全面落实“一企一策”、“互保共建”等措施，支持企业扩大生产，加快技术改造和新产品研发，加大中小企业培育帮扶力度，推动工业经济企稳回升，全市规模以上工业增加值完成102亿元，增长11.5%。

毫不放松“三农”工作，积极应对冬春连旱及洪涝、冰雹等自然灾害，及时安排救灾资金，支持恢复农业设施，协调扩大政策性农业保险，引导群众调整结构、稳粮扩经、发展畜牧，最大限度减轻灾害损失。全市推广旱作农业技术246.4万亩，粮食总产111.3万吨。牛饲养量104万头、出栏41万头，果品、蔬菜产量分别达到100万吨和124万吨。农业增加值完成77亿元，增长6.5%。

认真落实促进消费政策措施，培育扩大消费需求，优化消费环境，实施了平凉国际商贸城、陇东农副产品批发交易中心、金果博览城等一批大型商贸物流项目，建成8个标准化乡镇农贸市场和10个农村商品配送中心，城乡商贸流通体系不断完善，居民消费价格总水平控制在103.2%，社会消费品零售总额增长14.5%，消费拉动生产总值增长6个百分点。

（二）努力调结构，转型发展迈出坚实步伐

着眼提高经济发展质量，大力优化结构、延伸链条、促进转型，区域首位产业、战略新兴产业、富民主导产业扩量提质步伐加快，三次产业结构调整为23∶42.7∶34.3。

深入推进能源综合开发，设立了市级地质勘查基金，《灵台矿区总体规划》通过国家发改委初评，邵寨煤矿井筒见煤，五举、赤城煤矿和崇信电厂集煤站、聚丙烯等项目进展良好，华泓汇金煤化工及华亭电厂二期、安家庄煤矿、神裕河至邵寨矿区铁路分别取得国家、省发改委“路条”，红河油田新增探井90口，生产原油15.6万吨，煤炭就地转化率提高1.5个百分点。新兴产业培育和传统产业改造升级步伐加快，虹光高频无极灯一期、正大饲料一期、庆华建筑陶瓷等项目建成投产， 2.8兆瓦光伏电站并网发电，碳纤维产业园积极推进，非煤工业增加值在规模以上工业增加值中的比重提高5.1个百分点。

创新农业经营模式，强化政府引导、企业带动和科技支撑，积极推广新优品种、先进种养技术和高效生产模式，建办了一批产业基地和示范园区。全市新建改扩建龙头企业33户，规范注册农民专业合作社461个，流转土地10.2万亩、林地13万亩，组建家庭农场6个，培育百亩以上规模经营大户668户，建成规模化养牛小区56个，其中万头养牛场4个，落实果园标准化基地120万亩，发展百亩以上设施蔬菜示范点30个。

抢抓建设华夏文明传承创新区机遇，着力打造“神奇秀美崆峒山、天下养生第一地”，启动实施了以崆峒山生态文化旅游示范区为龙头、以六大特色文化主题区块和三大文化工程为主要内容的“163”计划，实施文化产业项目67个，新增文化企业137家，文化产业增加值增长30%。实施重点旅游项目36个，在央视等高端媒体和周边23个主要客源城市强化宣传推介，平凉旅游知名度进一步提升，旅游接待人数、综合收入分别增长26.3%和26.5%。以文化旅游产业为带动，交通、商贸、金融、房地产、信息等服务业较快发展，第三产业增加值增长9.4%。

（三）强力夯基础，城乡面貌显著改善

按照“一中心两园区”城市发展布局，中心城市集中实施重点项目84个，完成投资75.2亿元。全力实施热电联产集中供热续建工程，供暖面积达到500万平方米，关闭燃煤锅炉112座，有效减少了烟尘排放和大气污染，提高了城区供热质量，改善了人居环境；完成土地征用6260亩、房屋征收18.7万平方米，东大门改造一期、平沿路中段、双桥路泾河大桥建成通车，柳湖路东拓、双桥路北拓、纵三路延伸线、东大门改造二期等道路工程及绿地公园二期、“八沟一河”汛期隐患综合治理、雨水系统改造等加快实施；及时有效开展拆违治乱，城市管理水平稳步提升。平凉工业园区“一纵一横”道路、南北路泾河大桥等基础配套项目进展良好，申报国家级经济技术开发区通过省上评估。完善规划，落实扶持政策，崆峒山生态文化旅游示范区建设积极推进，平凉原生态民俗文化体验区启动建设。全市规划体系进一步健全，中心城市、6县县城和重点小城镇扩容提质成效明显。全市城镇化率提高到33.15%。

持续推进新农村建设，整合项目资金，突出路水电、垃圾收集、绿化美化等基础配套，抓建城乡一体化示范乡镇3个、新农村建设试点村59个、“三清五改”示范村137个，完成投资21亿元，新农村试点村、“三清五改”示范村覆盖面分别达到44.8%和49.5%。

全力加快交通、水利、电力、通讯等设施建设，平凉机场与西部机场集团签订委托管理协议，西平铁路建成运营，天平铁路关山隧道顺利掘进，泾渗公路和崇白路、罗长辅道改造建成通车。配合完成了白龙江调水、引洮二期静宁受水区工程前期规划及评审，灵台新集水库通过黄委会初审，治理河堤178公里。330千伏灵台送变电工程建成投运。启动“智慧平凉”建设，信息技术在经济发展、公共管理、社会服务中的应用步伐加快。

（四）着力促创新，发展活力持续增强

创新金融工作，设立市长金融奖，引进交通银行设立平凉分行，商业银行新增县乡分支机构39家，发展小额贷款公司11家。组建了富民交通运输发展公司。成功发行我市第一只企业债券10亿元，全市直接融资45.8亿元；市投资发展、惠民、泓源公司和崆峒旅游集团完成融资33.4亿元，保障了“一中心两园区”重点项目资金需求。完善银政企联席会议制度，加大金融对“三农”、小微企业和重点项目支持力度，全市新增贷款60亿元，贷款余额增长22%；总行直贷13.66亿元。推进财政管理创新，优化支出结构，国库集中支付改革全面推行，公务卡应用改革推广到所有一、二级单位，市财政支持县区财力和投入中心城市建设资金分别达到2.6亿元和6.17亿元。

支持非公有制经济加快发展，制定了促进非公经济跨越发展的《意见》，落实重点非公企业“一对一”帮扶措施，积极协调解决资金、土地等方面难题，市场主体不断发展壮大，新增私营企业863户、个体工商户1.01万户，新增从业人员3.47万人，非公经济增加值达到136.4亿元，占生产总值的比重提高到40.7%。

大力推进科技创新，加强院地院企合作交流，实施国、省列科技计划项目30项，建立市级工程技术研究中心4个，支持企业研发新技术新产品12项，创建市级特色产业科技示范园区6个，争创甘肃名牌产品6个，全社会研究与试验发展经费支出占生产总值的1.4%，科技对产业升级和经济发展的支撑能力不断增强。

（五）大力促和谐，社会建设与管理全面加强

进一步完善社会保障体系，新农合、城镇居民医疗保险政府补助均由240元提高到280元，企业退休人员基础养老金月平均增加168.9元，失业保险金发放标准平均提高134元，城乡低保、农村五保供养标准分别提高15%、28%和7.7%，城乡社会保障覆盖面稳步扩大，社会救助和医疗救助制度进一步规范完善。实施了一批敬老院、社会福利院、社区日间照料中心和农村互助老人幸福院等养老服务设施项目，市老年养护院投入使用。

启动实施六盘山片区区域发展与扶贫攻坚规划，投资6亿元，实施了46个整村推进、6个整乡推进和崆峒区北部干旱山塬区片区开发等重点项目，以工代赈易地扶贫搬迁困难群众1623户8117人。深化双联行动，完善帮扶机制，各级帮扶单位筹措帮扶资金1.76亿元，实施产业开发、基础设施、公共服务等帮扶项目2050个，措办实事1.7万件，有效解决了贫困村发展中的突出困难和问题。全市完成减贫14.32万人，扶贫对象人均纯收入增速高出全省平均水平2个百分点。

统筹推进城乡发展，积极实施以城带乡、以工哺农，农村教育、医疗、文化事业较快发展，生产生活条件显著改善，城乡居民收入差距逐年缩小。推动外来创业者和务工人员融入社区，多形式提供服务保障，注重解决工作生活方面困难，形成了外来人员与本地人和谐相处、包容互助、共促平凉建设的良好局面。

着眼促进人与自然和谐发展，全面加强生态建设和环境保护，完成城乡造林30万亩，治理水土流失350平方公里，新修梯田27.7万亩。开发复垦整理土地12.9万亩，建成高标准基本农田11.8万亩。大力发展循环经济，严格落实产能退出机制和建设项目总量指标前置审核，加强矿区、油田、水域、大气等重点领域执法监管和综合治理，加快企业废水、城市生活污水处理工程建设，企业清洁生产和节能减排扎实推进，主要污染物排放量控制在省上下达目标值以内。

积极创新社会管理，建成城市标准化社区11个、农村社区894个，华亭、崇信和灵台县农村社区建设试验全覆盖创建有序推进。深入开展平安建设，完善社会治安防控体系，认真倾听群众呼声，妥善解决了企业改制、土地房屋征收、“两参”人员待遇等遗留问题，有效化解了社会矛盾。严格落实安全生产责任，深化“打非治违”专项行动和隐患排查整治，安全生产形势总体稳定。深入推进食药监管体制改革，加强日常监管，保障了群众饮食用药安全。

加大项目支持和资金投入，扶持民族乡村壮大产业、改善基础条件、发展社会事业，民族团结进步创建和崆峒区“两个共同”示范区建设成效明显。加强宗教政策法规宣传教育，和谐寺观教堂创建深入推进，宗教依法管理工作不断加强。统计调查、物价、档案、外事、侨务、地震、气象、人防、地方志、红十字、残疾人事业等取得新的成效。全市军政军民团结、民族宗教和顺、社会和谐文明的局面进一步巩固提升。

（六）竭力惠民生，人民生活水平不断提升

加大资金投入和协调督办力度，向全市人民承诺的10件19项惠民实事有17项完成或超额完成，2项正在加快落实。全市新建各类保障性住房9949套76.7万平方米，完成农村危房改造2.1万户；在16个重点小城镇和建制镇开建公租房1130套，主体竣工率89%；改造县乡道路115公里，新建通村硬化路652公里，开建乡镇客运站31个，新开通城乡公交班线13条；建成农村饮水安全工程34处，解决了18.75万人饮水安全问题，农村饮水安全普及率达到92.9%；建成农村沼气生态户4000户，为群众发放太阳灶6500台；严格落实强农惠农政策，发放各种补贴12亿元。全市财政用于民生的支出占到总支出的74.3%。

全面落实促进就业创业各项政策，多渠道开发就业岗位，城镇新增就业3.9万人。发放小额担保贷款3.14亿元，扶持6394人成功创业，带动就业9635人。帮助6536名就业困难人员实现就业、1198户零就业家庭实现再就业。在全省率先开展高校毕业生“支企”服务，安置应届普通高校毕业生就业7439人，就业率达到88.8%。输转城乡劳动力48.5万人，创劳务收入62亿元。

优先发展教育，投资7.85亿元，新建改扩建校舍44.2万平方米，新建改建幼儿园44所，建成农村义务教育学校食堂158个，建成了一批标准较高、配套齐全的中小学校，启动了中心城市学校布局调整。累计发放“两免一补”、普通高中、中职学生资助资金及生源地助学贷款4.74亿元，惠及学生34.5万人次。

加强医疗卫生服务体系建设，完成市级全科医生临床培养基地主体工程和20个乡镇卫生院改扩建、70个标准化村卫生室建设，3 个县级医院和市精神卫生中心住院楼、综合楼投入使用，在全省率先建立新农合市级统筹模式，基本药物零差率销售覆盖基层医疗卫生机构，农村居民政策范围内住院报销比达到77%。强化计生政策引导和服务管理，全国利益导向政策体系示范区创建加快推进，全市实现“国优”全覆盖，人口自增率控制在5.52‰。

加快发展公共文化事业，市广电新闻中心基本建成，宝塔梁综合开发规划方案通过初评，完成了灵台数字影院建设和泾川、华亭数字影院前期工作，建成乡镇、社区体育健身中心9个，“三馆一站”服务群众290多万人次，城乡文化生活更趋丰富。

各位代表，一年来，我们着眼提高政府公信力和执行力，积极推进职能转变和管理创新，加快建设清廉政府、服务型政府。进一步完善政府决策管理机制，修订了市政府工作规则及责任目标考核办法，制定了城市规划土地建设审查委员会议事规则。自觉接受人大、政协监督和社会舆论监督，实行市政府常务会议议定事项向人大报告、政协通报制度，积极配合人大、政协开展视察、调研，制定市政府办理人大代表建议和政协提案工作考核办法，建议提案办复率100%。深化行政审批制度改革，取消和调整行政审批事项64项，简化审批要件和审批程序，审批时效进一步提高。扎实推进依法行政，强化效能监察，推进政务信息公开，成立市公共资源交易管理委员会办公室，公共资源交易中心、政务服务中心、便民服务热线受理中心及网上审批系统、政务公开网站建成运行，并配套跟进了全程电子监察。认真落实中央及省、市委关于改进工作作风、密切联系群众的规定，坚持一切公务节约从简，严控各类节庆活动，精简会议文件，严格审批因公出国（境）考察，扎实开展公务用车、办公用房清理整顿，会议、文件分别压缩20%、15.5%，因公出国人员减少52%，“三公”经费压减12.2%。加大纠风治乱力度，加强重点领域、重大项目和领导干部离任审计监督、行政监察，以人、财、物管理为重点的廉政风险防控工作不断加强。

各位代表！过去一年的成绩来之不易。这是省委、省政府和市委坚强领导，市人大、市政协监督支持，全市广大干部群众共同奋斗的结果。在此，我代表市人民政府，向全市各族人民，向中央、省驻平单位和驻平部队、武警官兵，向各民主党派、工商联、无党派人士，向离退休老同志，向关心、支持平凉发展的各界人士，致以崇高的敬意和衷心的感谢!

与此同时，我们也清醒地看到，全市经济社会发展中还存在不少困难和问题，特别是去年地区生产总值、固定资产投资、社会消费品零售总额等主要经济指标增速与年初计划和省目标值有一定差距，这既有宏观经济下行的不利影响，也与政府自身工作有关。一是我市资源依赖型经济特征明显，产业发展层次不高，应对市场变化、抗御市场风险的能力较弱，尤其是受煤电市场波动影响，一些煤电企业产销增速减缓、效益下滑，直接影响到生产总值等计划目标的完成；二是体制机制还不适应加快转型发展的要求，部分行政职能分散、交叉、重叠，一些审批事项程序繁琐、时限冗长，土地、矿产资源、资本等生产要素市场化改革滞后，非公有制经济和现代服务业发展缓慢，经济发展内生动力不强，缺乏新的增长点和大项目支撑；三是极少数部门和干部创新意识不强，服务效率不高，推动落实不够有力，特别是重大项目谋划不足，一些项目前期工作不扎实，开工迟，进展慢，没有按计划建成发挥效益，还有一些谋划的事情由于资金短缺没有落实到位。这些问题，我们将进一步健全机制，强化措施，努力加以克服和解决。

2014年工作任务

2014年是深入贯彻落实党的十八届三中全会精神、全面深化改革的开局之年，也是完成“十二五”规划、加快转型发展的关键一年。当前，世界经济形势复杂多变、复苏缓慢，国内经济不确定因素较多、下行风险依然存在。但综合分析，我们也面临诸多机遇和有利条件。随着十八届三中全会《决定》的贯彻落实，各项改革深入推进，我们发展中面临的一些体制机制障碍将得到有效破解，发展活力将进一步增强；国家支持西部发展、建设丝绸之路经济带，为我们加快改善基础条件、承接产业转移、扩大开放开发带来了难得机遇；省委、省政府关于加快向西开发、支持华夏文明传承创新区建设、实施“3341”项目工程和“1236”扶贫攻坚行动等决策部署，为我们争取更多项目资金、发展壮大特色产业提供了政策支持。我们将进一步发挥比较优势，突出重点领域，凝聚各方力量，努力推动经济社会平稳较快发展。

政府工作的总体要求是：以党的十八大和十八届二中、三中全会精神为指导，深入学习习近平总书记系列重要讲话精神，全面落实中央、全省经济工作会和城镇化工作会议精神，按照市委三届七次全委扩大会暨全市经济和城镇化工作会议的决策部署及深化改革增活力、提质增效促转型、统筹兼顾惠民生、凝心聚力干到位的总要求，紧盯全面脱贫和建成小康社会两大目标，突出主题主线，牢牢把握稳中求进的总基调，把改革创新贯穿于经济社会发展各个领域各个环节，着力调整优化结构，着力强化项目建设，着力推进扶贫攻坚，着力统筹城乡发展，着力保障改善民生，着力建设生态文明，促进经济持续健康发展、社会和谐稳定，努力实现有质量、有效益、不留后遗症的发展，迈出小康和谐文明生态平凉建设新步伐。

经济社会发展的主要预期目标是：地区生产总值增长11%，规模以上工业增加值增长13%，固定资产投资增长26%，社会消费品零售总额增长14%，进出口总额增长10%，地方财政收入同口径增长12%，城镇居民人均可支配收入增长12%，农民人均纯收入增长14%，城镇登记失业率控制在4%以内，居民消费价格指数控制在103.5%以内，人口自然增长率控制在7.1‰以内，单位生产总值能耗和主要污染物排放完成省上控制指标。

围绕上述思路目标，着力抓好以下八个方面工作：

（一）全面深化改革，增强经济发展内生动力。认真落实中央和省上决策部署，抓好以经济体制改革为重点的各项改革，充分发挥市场在资源配置中的决定性作用，以改革增活力、促发展。

培育壮大市场主体。大力支持非公有制经济加快发展，推行工商注册便利化，取消最低注册资本限制，削减资质认定项目，逐步改注册资本实缴登记为认缴登记制；废除各种不合理规定和隐性壁垒，坚持“非禁即入”，引导民间资本进入基础设施、市政设施、社会服务等领域；深入实施“百强民营企业帮扶工程”，培育销售收入过5000万元非公企业20户、过亿元企业10户，非公经济增加值增长17.25%。引导在平央企、省属企业发展混合所有制经济。妥善解决企业改革遗留问题。

深化农村重点改革。加快构建新型农业经营体系，坚持家庭经营基础地位，大力发展专业大户、家庭农场、农业企业等新型农业经营主体。推进农村产权制度改革，建立新型农村用地制度，完成宅基地使用权证登记发放，加快集体林权制度配套改革，探索利用土地承包经营权、农民住房财产权、林权进行抵押担保。维护农民生产要素权益，保障农民工同工同酬、农民公平分享土地增值收益。健全农业支持保护体系。加快水利改革试点，推进水资源管理体制和水价机制改革。

促进金融创新发展。发展壮大商业银行及保险、证券、信托、投资、担保等金融业态，争取发行市投资发展公司二期企业债券10亿元以上。抓好上市资源企业培育，力争有2户企业进入上市辅导。加快农村信用社改制步伐，支持民间机构发起设立中小型金融机构。规范小额贷款公司管理，积极发展农村资金互助组织。加强惠民、泓源、富民交通、崆峒旅游等企业规范运营，加快组建水利投资发展公司，实现融资40亿元。

深化财税体制改革。完善预算管理制度，扩大部门预算公开范围，从紧从严控制一般性支出，严格执行人大审议通过的预算安排。加强绩效预算管理，规范国库管理改革，扩大公务卡应用覆盖面，启动国库集中支付电子化工作。扩大“营改增”试点范围。完善不动产统一登记制度，建立登记信息管理平台。落实中央防控债务风险政策措施，加强地方债务管理。

（二）加快转变发展方式，提升产业整体素质。抢抓国家支持资源型城市可持续发展机遇，优化产业布局，延伸产业链条，强化科技创新，促进优势产业规模扩大、结构优化、效益提升。

大力推进新型工业化。围绕打造千亿级煤电化冶产业集群，加快延伸煤电化、煤电冶、煤电材产业链，煤电化产业增加值增长12%以上。改造提升传统产业，实施创新驱动和质量振兴战略，支持重点企业建立研发机构，加快技术改造和产品升级，完成新产品开发20项。加快培育新能源、新材料等新兴产业，力促碳纤维产业园和光伏产业园一期达产达标，生物胶原蛋白肽生产线建成投产。非煤工业增加值增长15%。加快建立中小企业公共服务平台，新增规上工业企业10家。加强工业园区（集中区）基础设施建设，创新运行机制，平凉工业园区申报国家级经济技术开发区力争通过国家批复，加快华亭、静宁工业园区管理体制改革，全市新入园（区）千万元以上项目40个以上。

加快发展现代农业。深入实施现代农业“五个百万”增收工程，依法加强基本农田保护，重视加强粮食生产能力建设，扩大旱作农业技术推广，稳定增加粮食产量，确保粮食安全；加快推进牛果菜产业适宜区全覆盖，加大先进适用技术应用，落实肉牛冻配改良、秸秆转化利用和果园标准化管理、设施蔬菜种植等措施，建成标准化养牛小区20个，新植果园28万亩，发展蔬菜生产集中区15处，新增无公害、绿色、有机和地理标志认证农产品生产基地10个。加大疫病防治、质量检测、品牌保护力度，扩大农业保险承保规模和覆盖面，培育壮大农产品加工、贮藏、运销业，发展农民专业合作组织100个，新建改建龙头企业25户，加快构建产加销有机结合的高效农业产业体系。

壮大提升服务业。开工建设公铁物流园，推进平凉国际商贸城、陇东农副产品批发交易中心、金果博览城等大型商贸综合体建设。完善市场布局和流通链条，新建一批社区放心粮油、蔬菜便利店和连锁超市，加快县区配送中心、乡镇农贸市场和村级农家站三个全覆盖步伐。大力发展社区养老、家政服务、养生保健等生活性服务业和现代物流、融资租赁等生产性服务业，支持发展教育培训、信息中介、文化创意、电子商务等新兴服务业，促进以服务业为重点的第三产业增加值增长11.5%。

（三）强力推进项目建设，切实增强发展后劲。坚持扩大总量与优化结构并重，深入实施“3341”项目工程，以“十大工程”为统揽，全力抓好招商引资和项目建设，着力夯基础、强产业、增后劲。

精心谋划论证项目。准确把握产业发展方向和投资导向，深入论证、超前谋划项目，全市储备项目投资额动态保持在4000亿元以上。加大项目前期投入和汇报衔接力度，加快酒钢煤转化循环经济、平定兰铁路、宝中二线、宝麟铁路北延线、固原至平凉城际铁路、静庄高速等项目前期进度，力促安家庄、唐家河煤矿、华亭100兆瓦光伏发电、平凉支线机场、引洮二期静宁受水区、崆峒水库改扩建、灵台新集水库、330千伏静宁变电站等项目前期工作取得实质性进展，完成华亭电厂二期、白平铁路静宁段、华崇灵二级公路等前期工作，争取有更多项目早日开工。

扩大招商引资规模。大力推行企地联合招商、产业链招商、园区招商和以商招商，瞄准全国、行业、民营500强，争取大型企业在我市建立区域总部、研发中心、营销中心和生产基地，加强与行业协会、专业协会、各地商会洽谈合作，力争招商引资签约规模达到500亿元。进一步健全完善招商引资项目推进落实机制，加强签约项目对接落实和跟踪服务，招商引资到位资金增长25%以上。

加快重点项目建设。坚持基础设施先行，力促天平铁路年内建成，开工建设平凉至天水、华亭至大桥村高速和平华一级公路，争取实施国道312线改造提升工程；新建平凉客运枢纽站和快速客运站，建成平凉道路运输应急救援指挥中心；实施泾河、葫芦河河堤治理等水利工程70项；建设330千伏尚家塬变电站，力争实施平凉750千伏开关站扩建工程；推进“智慧平凉”建设，加快光网城市和4G网络建设。大力推进能源化工项目，力争邵寨煤矿形成综采工作面，五举、赤城煤矿基本建成主副风井；加快红河油田百万吨产能会战、聚丙烯等项目建设，华泓汇金煤炭深加工基地通过国家核准并开工，陇能煤转化循环经济部分项目开工实施，建成平凉天元煤电化科技研发及生活基地；完成西气东输三线工程泾川段建设。开工建设平凉煤矿机械加工制造项目，加快天纤棉业二期等项目建设。抓好正大饲料二期、秦宝万头现代肉牛产业园等农业产业化项目建设。

强化项目保障服务。完善项目建设推进及协调保障机制，实行市级领导分工联系重点项目，落实动态管理、定期通报、跟进督办制度。依法依规做好土地房屋征收，建立项目用地投资强度评估监管机制，合理安排各类建设用地。加大信贷支持力度，激活民间投资，扩大市场化融资，多渠道筹措建设资金。优化项目建设环境，建立重大项目审批“绿色通道”，提供便捷高效服务，确保各类项目快推进、早建成。

（四）打好扶贫攻坚战，加快脱贫致富步伐。以增加贫困人口收入为核心，以关山林缘区、干旱山塬区、黑河流域和民族乡村为主战场，集中实施“1236”扶贫攻坚行动，全年减贫12万人。

持续改善生产生活条件。加快实施六盘山片区区域发展与扶贫攻坚规划，集中抓好45个整村推进项目。争取国开行贷款支持，加快行政村通畅工程、农田水利建设和农村电网改造，抓好农村危旧房改造和饮水安全工程建设，加强农村公路管理养护，贫困村动力电覆盖率达到95%以上，自来水入户率达到96%以上，70%的村通硬化路。建成易地扶贫搬迁工程安置点21个，搬迁贫困群众1328户，配套完善教育、卫生、文化等公共服务设施。

大力培育富民产业。支持贫困乡村发展牛果菜、中药材、脱毒种薯、苗木繁育等特色产业，增加农民生产经营性收入。加强信息服务、技能培训和就业指导，扩大有组织劳务输转，实现劳务收入59亿元。

着力提高扶贫对象技能素质。以“两后生”、劳务工为重点，分类制定教育培训计划，加大种养技术、务工技能培训力度，完成劳动力培训20万人次，实现贫困县区职业技能培训和职业技能鉴定全覆盖。加大贫困地区基础教育投入，研究制定贫困家庭学生教育扶助政策，通过增强下一代文化科技素质，消除贫困代际传承，帮助贫困群众“拔穷根”。

创新扶贫开发工作机制。整合项目、资金，拓展融资渠道，引导金融机构扩大对贫困地区信贷投放，有效解决贫困村发展资金短缺问题。深入推进双联行动，实行双联绩效与政绩考核挂钩，加快推进“八个全覆盖”，全力帮助贫困乡村改善条件、发展产业。

（五）加快新型城镇化建设，推进城乡发展一体化。围绕破解城乡二元结构，加快发展以人为核心的新型城镇化，推动中心城市、县城、小城镇和新农村功能互补、协同发展，城镇化水平提高2个百分点。

完善城乡规划体系。按照新型城镇化发展要求，启动市域城镇体系规划编制，高标准完成第四轮《平凉市城市总体规划》修编。围绕“一中心两园区”城市布局，修订中心城市建设控制性详规，加快编制历史文化保护规划、城市综合防灾规划，有序推进重点区域城市修建性详规编制。全面完成县城风貌规划和重要专项规划，开展重点小城镇总体规划、村庄规划修编，完善提升规划体系。强化规划执法检查，杜绝乱修乱建、抢修抢建等问题。

着力提升中心城市品质。统筹生产、生活、生态空间，深入开展中心城市建设提质提速年活动，集中实施“六提升四配套”工程，改造提升崆峒大道、柳湖路等“九纵十一横”主要街区；统筹推进泾河水景、堤岸景观建设；全面完成西寺街、过店街等11个棚户区改造，启动大岔河桥、保丰路等6个片区棚户区改造；实施绿地公园续建、虎山公园等城市绿化景观提升工程；加快东大门改造二期、柳湖路东拓、东湖路改造等工程建设，打通新科路、青年路等断头路，形成东西畅通、南北贯通的路网框架；启动盘旋路、火车站等6处节点改造，开建绿地公园两侧商业综合体；加快推进热电联产集中供热、雨污管网改造、城区防洪等配套工程，提升城市品位。

统筹推进县城、小城镇和新农村建设。加快户籍制度改革，促进有能力在城镇稳定就业、生活的常住人口有序实现市民化，支持崆峒区、华亭县、崇信县、平凉工业园区大幅提高城镇化率。持续推进县城、小城镇扩容提质和基础设施配套，扩大城镇集中供热覆盖面，提高污水、生活垃圾无害化处理率。培育城镇产业，支持建成一批工业带动、资源开发、商贸流通、旅游度假型示范城镇。抓建市列城乡一体化试点乡镇7个，加快新农村建设组团发展，实施新农村建设试点村20个、“三清五改”示范村200个。

切实加强城镇管理。坚持绿化、美化、净化、亮化同步推进，全力组织开展城镇精细化管理活动，打造整洁美丽文明、宜居休闲养生的平凉城市新形象。实施绿山、绿路、绿城、绿村、绿宅“五绿”工程，推进城乡住宅小区和单位庭院绿化全覆盖，构建中心城区泾河沿岸南北面山生态景观系统。强化综合执法，更新完善市政、环卫设施，落实单位门前“五包”责任制，加强城镇街路、居民生活区及背街小巷卫生保洁，推进环境卫生、市容市貌、交通秩序、市政设施网格化管理。抓好312国道沿线重要节点、市县所在地及近郊乡镇环境治理，下功夫整治脏乱差，提升整体形象。

（六）突出打造养生品牌，加快华夏文明传承创新区建设。抢抓国家建设丝绸之路经济带的重大机遇，全力实施全市华夏文明传承创新区“163”计划，抓好旅游大景区规划建设，加快建设中华崆峒养生地，推动文化旅游养生融合发展，打造“神奇秀美崆峒山、天下养生第一地”品牌。

推进平凉大景区建设。突出文化、生态等要素，抓紧编制全市大景区规划及崆峒山、大云寺两个核心景区建设规划，抓好崆峒山旅游配套设施二期、平凉原生态民俗文化体验区、大云寺二期等重大文化旅游项目建设，支持崆峒山生态文化旅游示范区和大云寺景区率先发展。加强宣传推介和旅游线路对接，创新市场运作模式，建设平凉旅游综合信息资源库。旅游综合收入增长20%。

加快养生平凉建设。以国家中医药养生保健旅游创新区建设为契机，把养生产业作为带动结构调整、转变发展方式的龙头产业，研究挖掘养生文化，统筹各类生产要素，集中打造六张养生名片，着力培育十大养生产业，加快实施十大配套工程，大力推广国医理疗、国医保健、运动养生等养生服务。重点以崆峒山生态文化旅游示范区为依托，多层次引进建设一批养生产业项目，打造集道文化、休闲养生、武术健身、旅游观光为一体的崆峒山道文化养生园，年内完成“中华崆峒养生地”总体规划编制和评审，全面启动建设并建成一批核心体验项目。

培育壮大文化产业。整合文化、旅游、养生资源，扶持建办一批手工文化产品、民间工艺品、地方特色文化旅游纪念品生产销售企业。加大文化产业招商引资，抓好签约项目落地实施。加快“文化集市”建设，建立农村文化产业贸易服务营销体系，促进农村文化产品集聚和产业发展。完成文化产业投资5.6亿元，培育产值上千万元文化企业5家，文化产业增加值增长20%。

统筹发展文化事业。完善文化管理体制，推进国有经营性文化单位转企改制，支持图书馆、博物馆等公益性文化事业单位组建理事会。实施宝塔公园建设工程，改造提升市文化馆、市图书馆，建设市美术馆、“非遗”中心，提高“三馆一站一屋”免费开放水平，抓好数字影院和“乡村舞台”建设，构建市、县、乡、村四级公共文化服务网络。开展 “平凉文化年”系列活动。实施重大文物保护工程，加快崆峒山世界自然、文化双遗产和皇甫谧针灸术世界非物质文化遗产申报工作。

（七）加强生态环境保护，推进生态平凉建设。坚持绿色低碳清洁发展，加快创建国家级生态示范市，构建生态安全屏障，提高可持续发展能力。

健全生态文明制度。坚守生态红线，建立生态文明目标体系和决策评价制度，严把资源开发利用关和环保安评关。建立生态文明管理保护和生态修复治理制度，落实资源有偿使用和生态补偿制度，扩大资源税征收范围，探索形成多元化生态投入保障机制，强化生态环境监测预警。建立生态文明考核问责制和生态环境损害责任终身追究制，实行领导干部自然资源、资产离任审计。

加强自然生态系统保护修复。推进天然林保护、三北防护林、退耕还林等重点林业生态工程建设，实施国家重点生态功能区转移支付项目，抓好城镇面山、城市和主干道沿线绿化，完成造林封育23.2万亩。加快巩固退耕还林成果口粮田、农综开发水保、坡耕地水土流失综合治理等工程建设，新修梯田24万亩，治理水土流失220平方公里，治理河堤110公里。实施陇东百万亩土地整治项目，开发整理土地10万亩。加强矿区塌陷区恢复治理，严格河道采砂和地下水开采管理。

狠抓治污减排。严格项目准入，杜绝新上污染项目，实施重点企业和规模化畜禽养殖企业减排项目86个。建成海螺水泥窑处理城市生活垃圾项目和庄浪、崇信县城区污水处理厂。推进大气污染防治，抓好燃煤锅炉脱硫改造和平凉电厂电除尘改造，有序关停中心城区热电联产集中供热区域燃煤锅炉。抓好城市饮用水源地建设与保护，加强地表水污染防治。持续推进农村环境连片整治和面源污染治理，废旧农膜回收率达到80%以上。

促进资源集约节约利用。推进循环经济发展，加强节能降耗工作，实施工业节能技改项目20个，加快既有建筑节能改造和供热计量改革。实施最严格的水资源管理，加强用水总量控制，建设一批节水示范项目。严格保护耕地，坚持集约节约用地，开展城乡建设用地增减挂钩试点，实现耕地占补动态平衡。

（八）着力保障和改善民生，促进社会和谐稳定。把保障和改善民生作为政府一切工作的出发点和落脚点，加快发展社会事业，创新社会治理方式，着力提高公共产品供给能力和公共服务水平，让发展成果更多更公平惠及全体人民。

集中力量办好民生实事。1.改造县乡道路154公里；建成通村硬化路600公里。2.新建农村饮水安全工程40处，解决10万人饮水安全问题。3.新建保障性住房11750套；改造农村危房2万户。4.新建改扩建中小学校舍9万平方米、农村幼儿园7所。5.新建标准化村卫生室20个；开建市级儿童专科病区；为已婚育龄妇女提供免费蓝氧治疗服务。6.提高城乡低保和农村五保供养标准。7.建成平凉市崆峒区殡仪服务中心；建设市应急避难场所。8.更新中心城市公交车58辆；新建改造公交候车亭186座。9.配套完善中心城市环卫设施；建设人民广场人行通道。10.实施市县乡广播电视网络覆盖暨市县有线电视城域网双向升级工程。11.实施中心城市雨水系统改扩建和污水全收集管网建设工程。12.实施农户科学储粮工程，新增农户科学储粮1万户。

千方百计扩大就业。继续落实城乡低保家庭高校毕业生求职补贴，引导高校毕业生面向基层和非公企业就业，应届普通高校毕业生就业率保持在85%以上。做好零就业家庭、困难家庭、复转军人和残疾人就业帮扶工作，深入推进全民创业行动，城镇新增就业3.2万人。

提高社会保障水平。加强灵活就业人员、非公经济从业人员、城镇困难人员等重点群体社会保险扩面工作，稳步提高城镇五项基本社会保险覆盖率，城乡居民社会养老保险参保率达到96%以上。开展城乡医疗保险统筹，推行城乡居民大病医疗保险。完善和落实被征地农民、村干部养老保险制度。抓好城乡低保清理规范工作。关心农村空巢老人、留守妇女和儿童。

促进社会事业协调进步。深化教育综合改革，努力促进教育公平；推进义务教育学校标准化建设，促进县域义务教育均衡发展；优化调整城区学校布局，着力解决择校热和大班额问题；加快发展学前教育；大力发展职业教育，开建平凉职业技术学院，加快医专升本步伐。深化公立医院改革，创新新农合支付方式，合理控制医疗费用；突出中医特色，健全基层中医药服务网络，提高基本公共卫生服务均等化水平。坚持计划生育基本国策，启动实施“单独两孩”政策。加大科技投入，鼓励创造发明，扩大和深化院地院企合作。开建市体育中心，广泛开展全民健身活动，提升竞技体育水平，组织好第十三届环青海湖国际公路自行车平凉段比赛。

创新加强社会治理。坚持系统治理、依法治理、综合治理、源头治理，深入推进平安平凉建设，进一步完善治安防控体系，强化重点区域、重点人群管理，严厉打击各类违法犯罪活动，增强人民群众安全感。健全完善重大决策社会稳定风险评估机制。建立畅通有序的诉求表达、矛盾调处和权益保障机制，及时妥善解决各类矛盾纠纷。依法加强网络管理，强化信息监管和舆情监测应对。加强重点领域、重点行业安全监管和隐患排查整治，有效防范安全生产事故发生。探索建立新型社区管理体制，新建城市标准化示范社区9个。加强社会主义核心价值观教育和公民思想道德建设，教育和引导群众自觉遵纪守法，提高社会文明程度。强化市场秩序整顿和产品质量监管，健全完善“四位一体”监管体系，建立覆盖全过程的监管制度，确保群众饮食、用药安全。落实防灾减灾措施，完善灾害救助体系，健全突发事件应急管理机制。做好国防教育和“双拥”工作。依法规范宗教事务管理，推进“两个共同”示范区建设，支持民族乡村加快发展。

加强政府自身建设

各位代表，面对今年繁重艰巨的改革发展任务，我们将以改革创新的精神，加快转变政府职能，扎实开展党的群众路线教育实践活动，着力推进治理体系和治理能力现代化。

深化行政体制改革，推进政府职能转变。认真贯彻落实党的十八届三中全会精神，深化政府机构改革，创新施政理念和管理方式，着力推进政企分开、政资分开、政事分开、管办分开。优化组织结构，理顺部门职责，加强发展战略、规划、标准的制定和实施，强化政府对经济活动的引导和规范。深化行政审批制度改革，进一步清理规范审批事项，简化审批程序，推行行政审批事项集中办理模式，推进审批事项网上办理及办理过程电子监察，提升公共资源交易平台运行水平，落实首问负责、限时办结、公开承诺和责任追究制，提升政府服务质量和效能。

规范施政行为，全面推进依法行政。牢固树立法治理念，着力建设法治政府。健全完善重大决策专家论证、公众参与、合法性审查和集体讨论决定等制度，提高依法、科学、民主决策水平。整合执法主体，相对集中执法权，推进综合执法，规范自由裁量权，加强行政执法监督，着力解决权责交叉、多头执法问题。认真落实市人大及其常委会决议决定，自觉接受人大、政协监督和社会舆论监督，健全机制、规范程序，加大人大代表建议和政协提案办理力度。深化政务公开，完善政府新闻发布和信息公开制度，保障人民群众知情权、参与权、监督权。

落实中央、省市委各项规定，大力转变工作作风。坚持领导带头，认真搞好党的群众路线教育实践活动，大兴求真务实之风，加强调查研究，改进文风会风，精简会议文件，坚决整治形式主义、官僚主义、享乐主义、奢靡之风。大力弘扬钉钉子精神，细化量化目标，分解靠实任务，切实做到事有人担、责有人负，定了就干、一抓到底。建立健全政府绩效评价和经济社会发展综合评价体系，加强经济运行分析调度，强化重大项目、重点工作跟踪督查，以踏石留印、抓铁有痕的决心，全力推动各项工作落实，诚心诚意为人民谋利益。

坚持从严治政，努力建设廉洁政府。严格落实党风廉政建设责任制，扎实推进惩治和预防腐败体系建设，健全风险预警和权力运行监控机制。加强对工程建设、土地管理、产权变动、公共资源拍卖、政府采购的行政监察和审计监督，依法治理和纠正损害群众利益的不正之风，严肃查处各类违纪违法案件。坚决落实《党政机关厉行节约反对浪费条例》和《党政机关国内公务接待管理规定》等规章制度，严格执行领导干部工作生活保障制度，扎实搞好党政机关办公用房清理整改，严格控制和压缩“三公”经费，树立清正廉洁的良好形象。

各位代表！新的征程蕴含新的希望。让我们在党的十八大和十八届三中全会精神指引下，在市委的坚强领导下，坚定信心，同心协力，改革创新，攻坚克难，全面完成今年经济社会发展各项目标任务，加快转型跨越，促进科学发展，为建设小康和谐文明生态平凉而努力奋斗！
